# *Mycobacterium chelonae* outbreak investigation at a quaternary pediatric hospital following the opening of a LEED-certified critical care tower: where does water sustainability intersect with infection control?

**DOI:** 10.1017/ice.2025.10344

**Published:** 2026-02

**Authors:** Andrea L. Ankrum, Silvia M. Caceres, Michael Torsell, Elaine Epperson, Vinicius Calado Nogueira de Moura, Jennifer J. Gilick, Michael Strong, Qingyun Liu, Matthew J. Strand, Rachel N. Wilsey, Jennifer R. Honda, Jane E. Gross, Felicia A. Scaggs-Huang

**Affiliations:** 1 Infection Prevention and Control, Cincinnati Children’s Hospital Medical Center, Cincinnati, OH, USA; 2 Department of Medicine and Pediatrics, National Jewish Health, Denver, CO, USA; 3 Department of Pediatrics, University of North Carolina, Chapel Hill, NC, USA; 4 Center for Genes, Environment and Health, National Jewish Health, Denver, CO, USA; 5 Division of Biostatistics, National Jewish Health, Denver, CO, USA; 6 Department of Cellular and Molecular Biology, School of Medicine, University of Texas Health Science Center at Tyler, Tyler, TX, USA; 7 Division of Infectious Diseases, Cincinnati Children’s Hospital Medical Center, Cincinnati, OH, USA; 8 Department of Pediatrics, University of Cincinnati College of Medicine, Cincinnati, OH, USA

## Abstract

**Objective:**

Investigate the increased incidence of *Mycobacterium chelonae* positive respiratory cultures in hospitalized patients.

**Design:**

Apply the Healthcare-Associated Links in Transmission of Nontuberculous Mycobacteria (HALT NTM) toolkit to an outbreak investigation of *M. chelonae.*

**Setting:**

Quaternary-care pediatric hospital and medical center in the United States with a recently opened LEED-certified critical care tower.

**Patients:**

Adult and pediatric patients with *M. chelonae* positive respiratory cultures between June 2022 and January 2024.

**Methods:**

An epidemiological investigation involving clinical and laboratory practices, water management, building construction and renovation projects. Environmental sampling of air vents, water sources and endoscope reprocessing equipment was performed. *M. chelonae* isolates recovered from patients and the environment were analyzed using whole genome sequencing and compared for relatedness.

**Results:**

Three clusters of matching environmental and patient isolates were identified. The most common environmental source of *M. chelonae* was ice/water dispensers with 40% positivity of sampled units. The critical care tower’s water system performance and metrics were suboptimal, leading to four physical remediation activities along with a hyperchlorination treatment.

**Conclusions:**

Recent and ongoing construction along with the implementation of a LEED-certified, low-flow water system in a new critical care tower provided enhanced opportunities for *M. chelonae* exposure at point of use locations such as ice/water dispensers. More national infection prevention and control guidance is needed to address the infection risks from water sustainability efforts and construction activities in healthcare facilities.

## Introduction

*Mycobacterium chelonae* is a rapid-growing nontuberculous mycobacteria (NTM) species, most frequently associated with eye and skin/soft tissue infections.^1,2^ Infrequently, hospitalized patients develop *M. chelonae* catheter-related and postsurgical infections after implants, transplants, and injections. Invasive infections, such as bacteremia, occur rarely in immunosuppressed patients.^1^ Pulmonary infections are also uncommon, even among patients with cystic fibrosis (CF); however, preexisting structural lung disease increases the risk of colonization and disease.^3^ Although NTM are increasingly a recognized cause of outbreaks and pseudo-outbreaks from hospital water systems, there are few published reports and limited national guidance on conducting these investigations and validated mitigation strategies are lacking.^4–7^ In recognition of this gap, the Healthcare-Associated Links in Transmission of Nontuberculous Mycobacteria (HALT NTM) study combines genomic and traditional epidemiologic investigation of healthcare-associated NTM outbreaks among people with CF receiving care in U.S. CF Care Centers but currently does not incorporate mitigation strategies.^8,9^ Here we describe an investigation utilizing the HALT NTM toolkit of an increase in *M. chelonae* in respiratory cultures in a pediatric healthcare system after the opening of a Leadership in Energy and Environmental Design (LEED)-certified critical care tower.

## Setting

The quaternary, pediatric hospital facility consists of three attached buildings - locations “A,” “B,” and the new 8-story critical care tower, “G.” “A” houses acute care inpatients along with hematology/oncology and respiratory chronic care units. “B” contains the operating rooms, same day surgery, procedure center and the postanesthesia care unit. “G” accommodates the inpatient pharmacy, clinical laboratory, sterile processing and decontamination (SPD), intensive care units (cardiac, neonatal, pediatric), and the bone marrow transplant unit, with each located on a separate floor. Construction of “G” was completed in September 2021 and was fully occupied by December 2021. Silver-level LEED 2009 Healthcare certification, which required a 20% reduction in indoor water use compared to baseline, was awarded in 2021. Strategies to reduce water use included installation of a low-flow system and water-efficient fixtures, such as low-flow toilets, faucets, and showerheads.^10^

## Methods

### Outbreak detection and case definition

**Figure 1.**
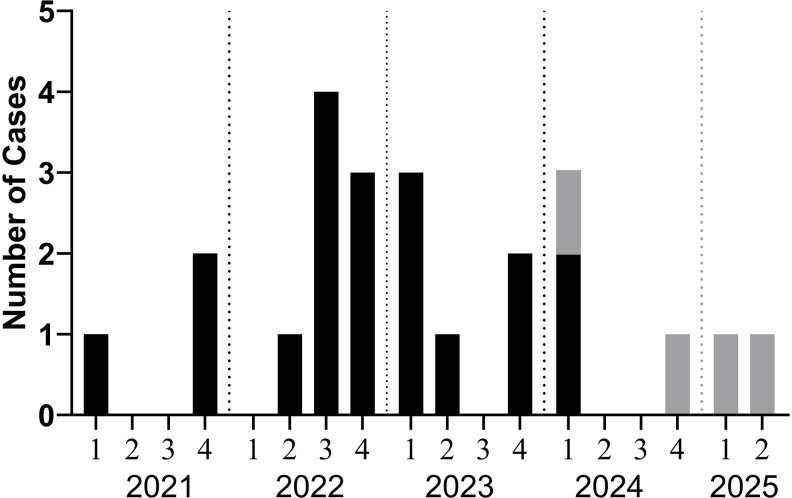
Incidence of *M. chelonae* from respiratory cultures by quarter from 2021 through the second quarter of 2025. The black bars represent cases that were part of the official investigation, and the gray bars represent cases that occurred outside the official investigation period.

In September 2022, the infection prevention and control (IPC) program detected an increase in incidence of *M. chelonae* recovered from clinical respiratory cultures via routine surveillance with five positive samples in four months. A retrospective review of clinical microbiology records uncovered only three *M. chelonae* isolates in the previous two years (2020–2021). An outbreak investigation using the HALT toolkit was initiated and reported to the local health department. The official outbreak investigation occurred from June 2022 to January 2024 and involved 16 patients, but continued surveillance revealed *M. chelonae* from respiratory cultures of 4 additional patients (Figure 1).

A case was defined as any inpatient or outpatient with *M. chelonae* isolated from a respiratory sample collected between June 2022 and January 2024. Clinical samples included bronchoalveolar lavage (BAL) or sputum.

### Respiratory microbiological and molecular testing

A majority of *M. chelonae* isolates were recovered incidentally from fungal media as acid-fast bacilli (AFB) cultures were not initially ordered. Identification of *M. chelonae* occurred in the clinical microbiology laboratory using matrix-assisted laser desorption ionization time-of-flight mass spectrometry (MALDI-TOF MS, bioMerieux Vitek MS, Marcy-I Etoile, France). Isolates were sent to National Jewish Health (NJH) Advanced Diagnostic Laboratories for antibiotic susceptibility testing and whole genome sequencing (WGS). Bacterial DNA was extracted using a modified method.^11^ The sequenced DNA was analyzed at the core genome level to determine genetic relatedness.^12^ Isolates were considered highly related and falling into a cluster using single nucleotide polymorphism (SNP) comparisons.^12^

### Healthcare environmental testing

Utilizing the Transmission and Acquisition of Nontuberculous Mycobacteria Outbreak Investigation (TrANsMit, NCT06155747) protocol, a virtual walk-through of the hospital was conducted on March 23, 2023, to develop an environmental sampling plan. From March 24th–April 10^th^, 2023, environmental water biofilm samples were collected from multiple sources (sinks, showerheads, ice/water units, drinking fountains) in the critical care tower (“G”), the perioperative suite (“B3”), and the Transitional Chronic Care unit (“A3”). Potable water and filters, air vents, endoscope reprocessing units and working channels in bronchoscopes were sampled in SPD. Samples were sent to the Environmental Nontuberculous Mycobacteria Reference Laboratory at the University of Texas Health Science Center at Tyler and processed as previously published.^13,14^ DNA from isolates identified as potential NTM was extracted and species identified by Sanger sequencing a 711 base pair region of the *rpoB* gene.^11,15,16^

### Epidemiological investigation

Electronic health records were used to create a line list of cases and capture demographics, diagnoses, dates of cultures, specimen type, surgical procedures, and specimen collection details. Case locations in the healthcare facility 18 months prior to *M. chelonae* positive culture including inpatient units, outpatient clinics, and operating rooms (OR) were collected. Based on common trends found among cases, interviews were conducted with multiple subspecialty care areas including pulmonology, otolaryngology, respiratory therapy (RT), anesthesia, perioperative services, and SPD to review clinical practices. The clinical microbiology laboratory was investigated to rule out contamination, media or reagent changes, and staff practices. Information from building maintenance was gathered on the design of the new critical care tower (“G”) as well as plumbing issues and water intrusions. Current and past construction projects were reviewed along with any related water and air handler outages.

### Genomic relatedness analysis

Respiratory and environmental *M. chelonae* isolates underwent DNA extraction and Illumina short-read WGS.^12^ Trimmed paired-end reads were mapped to the *M. chelonae* ATCC 19237 reference genome for variant calling.^17^ Genetic relatedness was assessed using core genome SNP differences with identification of clustered isolates based on a ≤ 30 threshold.^12,18^ Phylogenetic trees were created with the neighbor-joining method and 500 bootstrap replicates with MEGA11 and visualized using ggtree.^19,20^ WGS data are available at the National Center for Biotechnology Information (BioProject PRJNA319839).

### Statistical analysis

Comparison of proportions of fungal cultures from BALs was performed using a chi-squared test for equality of proportions using R (version 4.4.1) with significance determined at *p* < 0.05. Gannt Figure 5 and Supplemental Figure 1 were created using ggplot2 in R (version 4.3.2).

## Results

### Case characteristics

Cases included ten children and six adolescents/young adults, with a variety of complex chronic conditions (Table 1). Most cases were asymptomatic with respiratory cultures collected only for routine clinical surveillance, however four cases were treated with NTM-directed therapy, although it is unclear if *M. chelonae* was the primary source of pulmonary disease exacerbation (Table 2).

**Table 1.** Case characteristics including underlying medical conditions, age at time of culture and counts of facility visits and types of procedures in the 18 months prior to positive *M. chelonae* culture.

Case ID	Medical conditions	Age at time of culture (years)	Hospital days^a^ (total admissions)	“G” days^b^ (total admissions)	OR visits^c^	Outpatient visits^d^	Procedure types
Pt1	Myelomeningocele, type 1 laryngeal cleft, Chiari malformation	4	18 (6)	6 (2)	5	15	MLB, tonsillectomy, adenoidectomy, gastroscopy
Pt2	Subglottic stenosis, tracheomalacia	6	9 (2)	9 (2)	6	4	MLB, tracheal stoma revision
Pt3	Pulmonary hemorrhage, hilar mass	20	1 (1)	1 (1)	0	0	Bronchoscopy
Pt4	Bronchopulmonary dysplasia	0	279 (1)	157 (1)	3	0	Inguinal hernia repair, MLB
Pt5	Subglottic stenosis, tracheobronchomalacia	5	0 (0)	0 (0)	3	1	MLB, tonsillectomy, adenoidectomy
Pt6	Fanconi anemia, ventricular septal defect, duodenal atresia	23	20 (3)	11 (2)	1	4	MLB, flex sigmoidoscopy, NJ placement
Pt7	Schwachman Diamond Syndrome, bone marrow transplant	25	126 (2)	126 (2)	3	25	Bronchoscopy
Pt8	Myelomeningocele, Chiari malformation, chronic respiratory failure	4	21 (3)	2 (1)	5	20	MLB
Pt9	Cystic fibrosis, pancreatic insufficiency	11	19 (2)	1 (0)	2	23	Bronchoscopy
Pt10	Pallister-Killian syndrome, chronic respiratory failure	1	176 (4)	76 (2)	5	22	MLB
Pt11	Neurosarcoidosis, granulomatous lung disease	17	2 (1)	0 (0)	1	25	None
Pt12	Bronchopulmonary dysplasia, tracheomalacia	1	351 (1)	297 (1)	7	0	MLB, retinal laser procedure
Pt13	Atrial septal defect, trisomy 13, cerebral palsy	14	1 (1)	0 (0)	1	1	MLB
Pt14	Rett syndrome, chronic respiratory failure, laryngotracheal separation	18	239 (5)	0 (0)	9	7	Bronchoscopy, MLB, removal of pedicle screws, mediport insert
Pt15	Achondroplasia, airway malacia, restrictive lung disease	7	4 (2)	4 (2)	2	1	MLB, ear debridement, insertion of ventilator tube
Pt16	Spinal muscular atrophy, glass syndrome, arthrogryposis	7	45 (3)	14 (3)	1	9	Bronchoscopy

Note. OR, operating room, MLB, microlaryngeal bronchoscopy.

a Total number of hospital days 18 months prior to positive *M. chelonae* culture including the culture collection date if applicable. The total may represent multiple admissions.

b Number of hospital days in the “G” building 18 months prior to positive *M. chelonae* culture including the culture collection date if applicable.

c Number of OR visits 18 months prior to positive culture. Includes the day of culture collection if applicable.

d Total number of encounters 18 months prior to positive *M. chelonae* culture. Includes emergency department and clinic visits.

**Table 2.** Case and specimen characteristics.

***Case characteristics N = 16***	N	%
Mean case age in years: [range]	10.2 [10 months–25 years]	
Children (<1 to 11 years)	10	62
Adolescent/adult (14–25 years)	6	38
Gender:		
Male	9	56
Female	7	44
Hospitalized at the time of positive culture:	8	50
Underlying medical conditions:		
Immunosuppression^a^	3	19
Invasive ventilation	4	25
Chronic pulmonary disease^b^	13	81
Tracheostomy	11	69
Deceased^c^:	3	19
Anti-mycobacterial treatment:	4	25
Pulmonary endoscopy procedure in the six months prior to or including positive culture date:		
Flex bronchoscopy	15	94
Microlaryngoscopy and bronchoscopy (MLB)	11	69
Other procedures performed in the six months prior to or including positive culture date:	8	50
Specimen characteristics:		
Induced sputum	3	19
Bronchoalveolar lavage	13	81
Recovered from fungal culture	12	75
Acid-fast bacilli (AFB) smear-positive	1	6
Cases with follow-up AFB cultures:		
Obtained	7	44
Positive	2	12

a Defined as having a medical condition or treatment which suppresses normal immune system function such as cancer or a hematological disease.

b Defined as a history of disease which affects the pulmonary system and requires ongoing management.

c Determined by clinical team to be related to underlying medical condition and not *M. chelonae* positivity.

### Epidemiological investigation

The investigation revealed minimal temporal and spatial overlap between cases in inpatient units, outpatient clinics and ORs. Two cases had prolonged hospitalizations (>200 days) prior to positive *M. chelonae* culture (Pt4, Pt12). Another case (Pt3) was intubated prior to transfer from an area hospital and tested *M. chelonae* positive on the day of admission. Among the common procedures performed, 15 cases received at least one bronchoscopy, with a concurrent microlaryngoscopy in 10 cases. No common bronchoscope was used across all cases. Rigid scopes were not internally tracked; however, since the cases varied in age, we assumed that the same scope was not shared between all cases. Additionally, there was no significant overlap in surgical providers or procedure locations.

### Clinical investigation

Review of the clinical microbiology laboratory revealed no new practices or products during the investigation period. There was no significant difference in the number of fungal BAL cultures performed in 2021 vs. 2022 (*p* = 0.15). When comparing all NTM positive AFB BAL cultures between 2021 and 2022, there was a fourfold increase in unique *M. chelonae* positive cultures with no relative increase in all other NTMs, including species known to grow on fungal media (*M. abscessus, M. fortuitum*). Tap water was not used during routine clinical care (eg, RT) nor during surgical procedures, however for intubated patients with dry mouths, staff could provide ice chips or swabs moistened with tap water. In 2019, a bedside reprocessing procedure of Bivona™ tracheostomy tubes was implemented which included an initial rinse in tap water to remove secretions with the remaining steps using sterile water. Eleven of the 16 cases (68.7%) had a tracheostomy, with 10 of those cases having at least one hospitalization prior to their first positive culture where the tracheostomy tube may have been reprocessed. There were no sinks or other access to tap water inside the bronchoscopy rooms or ORs. Inpatient rooms contained hand hygiene and bathroom sinks and showers. Clinical supplies are not permitted within three feet of inpatient room sinks but in some patient rooms (“A”), the nursing supply cart is adjacent to the hand hygiene sink due to space constraints. In “G”, patient supplies are kept in a wall cabinet; however, staff may place patient care items in or around the sink basin. Ice/water dispensers for patients are located in inpatient nutrition rooms and family lounges.

### Environmental investigation and mitigation

Due to the association of cases with respiratory endoscopy procedures, scope reprocessing was reviewed including manual decontamination, automated high-level disinfection (hydrogen peroxide, peracetic acid, trisodium phosphate) and the drying process. Quality control documentation was found to be appropriate, and SPD staff could verbalize and demonstrate the processes for decontamination. No water shutdowns or plumbing issues occurred during the investigation period. Rarely, post-reprocessing, water would be found in a bronchoscope at the point of use. In January 2024, SPD implemented a secondary drying step involving forced air and visual inspection. In May 2025, new scope storage cabinets were installed with a constant air feature providing a continuous drying environment. These interventions have markedly reduced the incidence of wet scopes.

**Figure 2.**
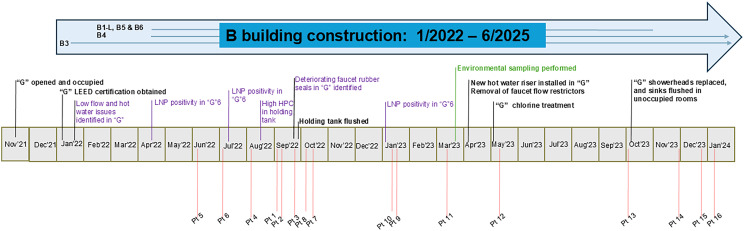
Timeline of outbreak investigation period (November 2021–January 2024). Building B construction is represented by the large blue arrow with the smaller arrows representing start and completion dates of projects on individual floors. Identified problems are purple. Environmental sampling is green. Respiratory *M. chelonae* cases are red lines with associated case numbers. LNP is *Legionella* non-pneumophila spp. “G6” is the sixth floor of the “G” building.

All construction projects requiring a water infection control risk assessment were reviewed along with data from IPC’s construction monitoring program (physical surveillance, *Legionella spp.* sampling and record-keeping audits). During the investigation period, no water outages or plumbing issues were reported in buildings undergoing renovation. However, the entire perioperative suite (“B3”) began extensive renovations in 2022 while continuing to occupy the same space (Figure 2).

Upon occupancy of “G”, low water pressures from inpatient unit sinks and patient showers were noted by staff and families. Bathroom showerheads allowed shower faucets to remain open, creating opportunities for stagnant water backflow. Hot water temperatures in several patient care areas did not meet the recommended target range (105°–120° F).^21,22^ In April 2023, an additional hot water return riser in “G” was installed and flow restrictors on faucets in medication rooms were removed. All showerheads and hoses were replaced and weekly five-minute hot water flushes for sinks in unoccupied rooms implemented in October 2023 to prevent water stagnation and improve water flow in pipes.

In examining the potable water system, “A” and “B” locations were supplied with city water via a shared line while “G” had a separate water line, with both lines connecting to the same city main line. The hospital hot water system was treated with copper/silver ionization as part of the water management plan. Routine testing included *Legionella pneumophila, Legionella* non-pneumophila (LNP), heterotrophic plate counts (HPC), chlorine levels and hot water temperatures (Table 3). In early 2023, LNP positivity was elevated in “G” and warranted a remediation of 12.5% chlorine disinfection of the potable water system.^23^ In August and September 2022, high HPC counts were detected in a water holding tank that supplied “A” and “B” locations. The city main line and the holding tank were flushed twice before HPCs were reduced back to acceptable levels. Water system issues and interventions are summarized in Figure 2.

**Table 3.** Routine water system testing control limits and corrective actions per the water management plan along with actionable results during the outbreak investigation period.

BUILDING DISTAL SITE SAMPLING^a^ Testing frequency: “G” quarterly, “A” and “B” biannually			
	**Control limits**	**Actionable results**	**Corrective action**
*Legionella spp.* (*L. pneumophila* and non-pneumophila)	< 10 CFU/ml <30% positivity	April 2022 “G” July 2022 “G” October 2022 “G” January 2023 “G” multiple locations	Flush and retest Flush and retest Hyperchlorination and super-heated flushing
Residual chlorine	0.2–2.0 PPM	N = 266 257 test points <0.2 PPM (97%) (range 0.0–0.44)	None
Hot water temperature	100°F–120°F	N = 268 33 test points <100°F (12%) (range = 70°F–123°F)	Evaluate and adjust mixing valve at each site

WATER HOLDING TANK Testing frequency: monthly			
	**Control limits**	**Actionable results**	**Corrective action**
Heterotrophic plate counts	< 500 CFU/mL	August 2022 (10,000 CFU/mL) September 2022 (3,000 CFU/mL)	Flush tank and main line, retest Flush tank and main line, retest

Note. CFU/mL, colony forming units/milliliter. PPM, parts per million.

a Testing occurs on each floor at distal points of use locations such as unit sink faucets.

### Environmental Microbiological investigation

**Figure 3.**
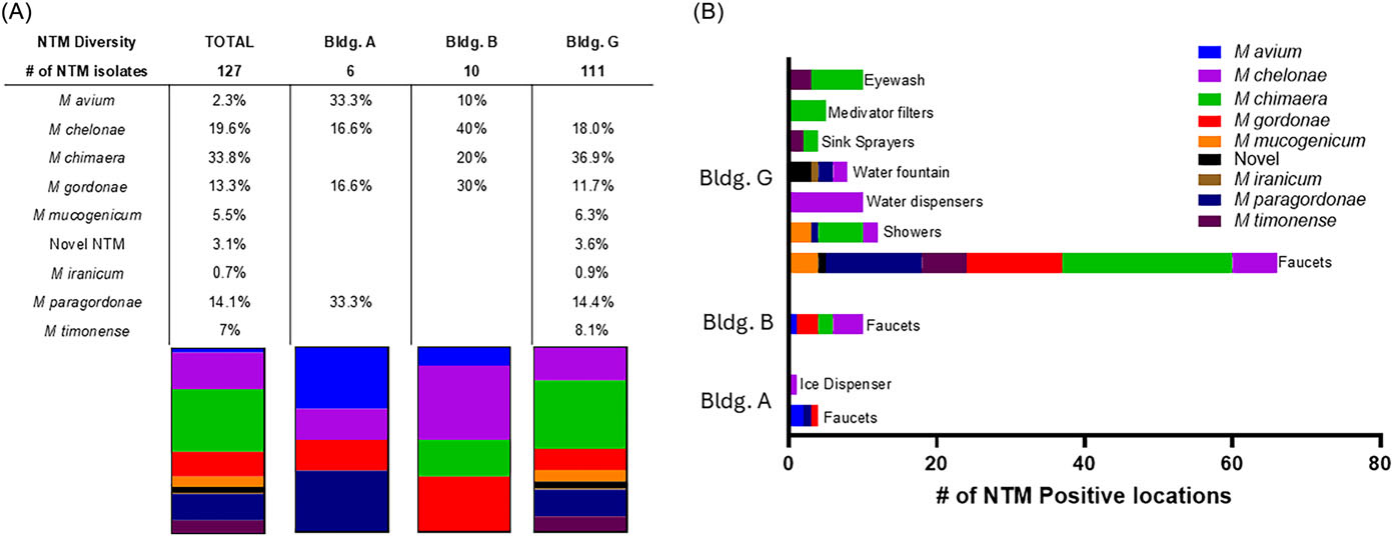
Environmental non-tuberculous mycobacteria (NTM) diversity. A. NTM diversity for each building. B. NTM environmental isolates by specific location. Certain locations may have recovered multiple NTM species.

A total of 380 healthcare environmental samples were collected. Of these, 21.6% (82/380) of samples from 17 source types were NTM culture positive (Figure 3). Eight different NTM species, as well as putative novel NTM, were isolated, of which *M. chimaera* (43/380 samples, 11.3%) and *M. chelonae* (25/380 samples, 6.5%) were the most frequently isolated. The 25 *M. chelonae* positive samples spanned 10 source types, most commonly in ice/water dispensers (10/25, 40%), unit entry sinks (3/25, 12%), and room sinks (3/25, 12%).

### Cluster identification

**Figure 4.**
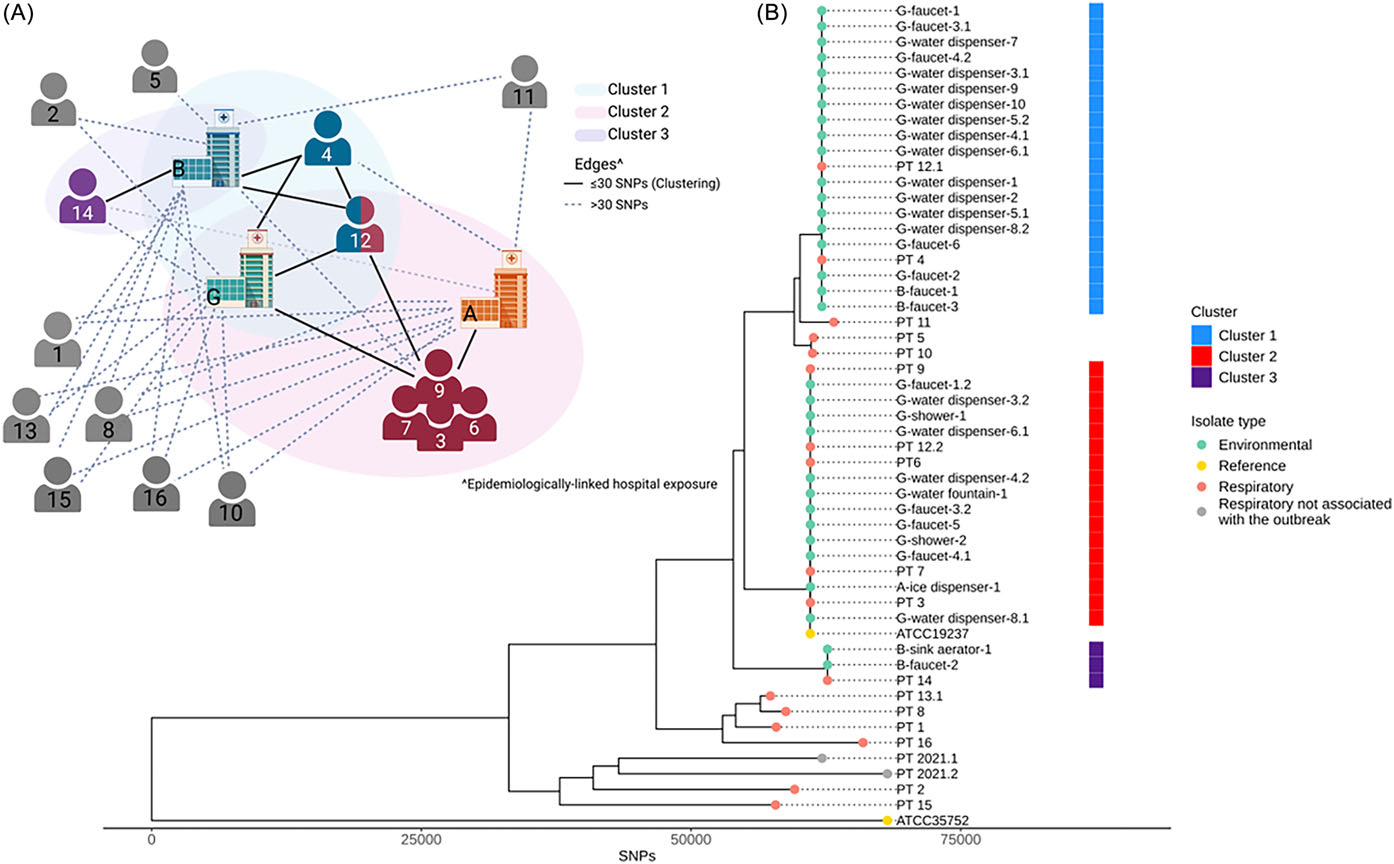
A. Epidemiologic cluster network analysis. Patients with clustered *M. chelonae* isolates are grouped by color (Cluster 1 is blue, Cluster 2 is red, Cluster 3 is purple, and patients with distinct unrelated infections are gray) and represented as a human torso. Patients with infections that had epidemiologic exposure to buildings are shown with a line connecting the patients and building [solid line represents matching patient-environment infections (≤30 SNP differences), dashed line represents unrelated patient-environment isolates (>30 SNP differences)]. *Created in BioRender. Caceres, S. (2025*) https://BioRender.com/ahiincb. B. Phylogenetic analysis of *M. chelonae* isolates. Environmental isolates are represented in green circles (labeled by building and specific location). Reference isolates, including type strains and previously published isolate genomes, are shown in yellow. Isolates from cases, not identified in a cluster or “unclustered,” are represented in gray circles (labeled by patient number). Clustered respiratory isolates are represented by salmon circles. Clustered patient and environmental isolates are color coded by thick bars to the right of the phylogenetic tree (Cluster 1 is blue, Cluster 2 is red, and Cluster 3 is purple). SNP: single nucleotide polymorphism.

**Figure 5.**
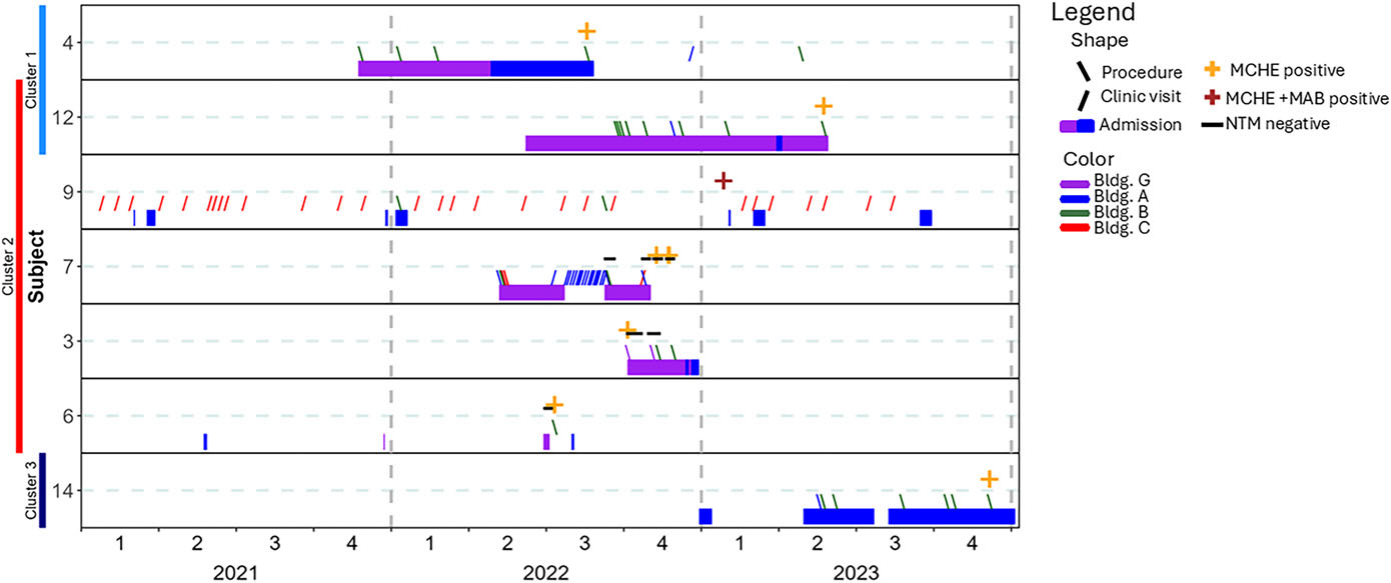
Timeline and location overlap analysis of cases with *M. chelonae* clustered isolates. Subject location is presented by building (G is purple, A is blue, B is green, and C is red) and location type within a building (clinic visits with forward slash, procedures with back slash, and hospitalization days with closed rectangles). Subject culture status is represented as *M. chelonae* culture positive (orange +), *M. chelonae* and *M. abscessus* culture positive (brown +), and NTM culture negative (negative dash). The clustered *M. chelonae* isolates are demonstrated by colored bars to the left of the timeline overlap graphic (Cluster 1 is blue, Cluster 2 is red, and Cluster 3 is purple). The number on the y-axis identifies the subject. The numbers on the x-axis represents time based on the year and quarter from 2021 through 2023. NTM = non-tuberculous mycobacteria; MCHE = *Mycobacterium chelonae*; MAB = *Mycobacterium abscessus*.

Genomic analysis of clinical and environmental samples revealed three *M. chelonae* clusters with 22 of 25 environmental isolates matching patients at the ≤30 SNP threshold (Figure 4). Cluster 1 contained isolates from two cases and 18 environmental locations, Cluster 2 contained isolates from five cases and 12 environmental locations and Cluster 3 contained isolates from one case and two environmental locations. One case (Pt12) had two strains of *M. chelonae* with one in Cluster 1 and the other in Cluster 2. Nine cases were not clustered with other cases or environmental samples. Of the isolates from 2021, one initially identified as *M. chelonae* was confirmed to be *M. franklinii* while the other two isolates were included in the analysis as references. Timeline overlap analyses for clustered cases (Figure 5) and unclustered cases (Supplemental Figure 1) highlight the common exposures to “G” and/or a procedure in “B”.

## Discussion

We describe the investigation into an increased incidence of respiratory *M. chelonae* cultures among pediatric and adult patients at a large quaternary-care hospital in the context of recent and ongoing construction and renovation. After an extensive epidemiological and environmental investigation utilizing HALT NTM methods, we hypothesized that construction of a LEED-certified critical care tower (“G”) along with renovations of an adjacent building (“B”), led to disruption of endemic water biofilms.^24^ Dispersion of these biofilms and the installation of a low flow water system may have facilitated *M. chelonae* colonization in the most distal, low flow, point of use locations such as ice/water dispensers, sink faucets and drinking fountains.^25^ Patient acquisition could have occurred through several routes including drinking water/ice, showering/bathing, tracheostomy tube reprocessing, contamination of patient care supplies around sinks and/or incompletely dried bronchoscopes.

Our experience demonstrates the complex and poorly defined relationship between maintaining a safe water supply for high-risk populations and environmental sustainability in healthcare settings. While the 2022 WHO Guidance for Climate-Resilient and Environmentally Sustainable Health Care Facilities calls for a less energy-intensive, waste-producing approach to healthcare, such as those promoted by LEED, there is limited guidance for IPC programs on how to manage the environmental infection risks these practices may introduce to vulnerable hospitalized patients.^26^ In our case, within three years of opening our critical care tower (“G”), four plumbing system remediations related to our water conservation efforts were required to improve water flow. Additionally, LEED-promoted low flow systems are known to reduce water use which indirectly increases water retention time allowing for chlorine dissipation and sediment buildup which can promote microbial growth, particularly NTM.^27–30^ After instillation of a LEED-promoted low flow system in our institution, the resulting low chorine levels along with our substandard hot water temperatures may have permitted growth of LNP. Detection of LNP ultimately led to mitigation via hyperchlorination of the “G” building after patient occupancy in addition to two treatments performed during construction. A similar scenario was reported by a hospital in Italy which hypothesized an increase in respiratory cultures with NTM was due to water supply colonization facilitated by previous hyperchlorination treatments.^31^ Since NTM have demonstrated resistance to chlorine disinfection, treating our water system potentially enhanced NTM growth at fluid-surface interfaces by eliminating competing chlorine-susceptible organisms.^32–34^

While NTM in potable water may not pose a serious infectious risk to healthy individuals, exposure of critically and chronically ill individuals to NTM increases the risk for acquisition and disease.^35–37^ Similar to our findings of genomically matched NTM isolates from cases and environmental water biofilms from ice/water dispensers, there are previously described NTM outbreak reports linking human NTM infections to environmental NTM from ice and drinking water.^38–40^ Prior studies have demonstrated that elimination of tap water, including ice and water from dispensers, for high-risk inpatients resulted in healthcare-associated NTM infection reduction.^35,37^ Compilation of outbreak findings into robust standardized studies such as HALT NTM may provide healthcare facilities with more uniform, consistent approaches to investigating, mitigating, and preventing healthcare-associated NTM outbreaks in high-risk populations. We are actively working on mitigation of our ice/water dispensers but have not yet identified the optimal remediation solution beyond discontinuing their use in our high-risk areas and susceptible populations.

Our outbreak investigation had several limitations. Negative test results are not indicative of the absence of NTM from environmental sources. Additionally, environmental sampling did not occur until 10 months after the first case of the outbreak limiting timely identification of potential healthcare sources for acquisition. However, there is strong evidence that NTM clones are stable in water biofilms for extended periods of time with a previous study demonstrating repeated recovery of NTM over a 41-month period from a hospital recirculating hot water system.^41^ Not all products or devices could be tracked at the patient-level, limiting our ability to further investigate the role of rigid scopes. Conducting a colonization point-prevalence survey of our entire patient population was not done due to feasibility and identifying additional cases would likely not have further aided our investigation as the data collected provided sufficient information to identify environmental sources.

In summary, national guidance on NTM environmental screening and water management in healthcare settings for human disease-causing NTM pathogens is urgently needed. Environmental NTM infection screening and mitigation guidelines should be developed in synchrony with current mandates for environmental screening and water management of *Legionella* infections as some *Legionella* mitigation strategies are known to be NTM enhancing.^33^ Additionally, environmentally sustainable programs to promote water reduction are essential to long-term water use plans but effects on growth of opportunistic pathogens, including NTM, should be studied before implementation in healthcare facilities. Understanding downstream consequences of LEED strategies will ensure healthcare centers are prepared to identify and mitigate potential infection risks among vulnerable populations. Environmental sustainability is a critically important goal in healthcare and IPC programs need to be prepared with knowledge of best practices when confronted with conservation efforts.

## Supporting information

Ankrum et al. supplementary material Ankrum et al. supplementary material

## Supplementary material

The supplementary material for this article can be found at https://doi.org/10.1017/ice.2025.10344

## References

[1] Akram SM, Rathish B, Saleh D. Mycobacterium chelonae infection. In StatPearls [Internet]. Treasure Island, FL: StatPearls Publishing; 2023. https://www.ncbi.nlm.nih.gov/books/NBK559302/ Accessed January, 2024. 28613557

[2] Hay RJ. *Mycobacterium chelonae*–A growing problem in soft tissue infection. Curr Opin Infect Dis 2009;22:99–101. 19276876 10.1097/QCO.0b013e328322b440

[3] Kendall BA, Winthrop KL. Update on the epidemiology of pulmonary nontuberculous mycobacterial infections. Semin Respir Crit Care Med 2013;34:87–94. 23460008 10.1055/s-0033-1333567

[4] Fraser VJ, Jones M, Murray PR, Medoff G, Zhang Y, Wallace RJ, Jr. Contamination of flexible fiberoptic bronchoscopes with *Mycobacterium chelonae* linked to an automated bronchoscope disinfection machine. Am Rev Respir Dis 1992;145:853–855. 10.1164/ajrccm/145.4_Pt_1.853 1554214

[5] Yoshida S. TM, Tsuyuguchi K, Suzuki K. Pseudo-outbreak of *Mycobacterium chelonae* caused by the water reservoir in a hospital. Jpn J Infect Prev Control 2009;24:109–112.

[6] Kressel AB, Kidd F. Pseudo-outbreak of *Mycobacterium chelonae* and *Methylobacterium mesophilicum* caused by contamination of an automated endoscopy washer. Infect Control Hosp Epidemiol 2001;22:414–418. 11583208 10.1086/501926

[7] Inkster T, Peters C, Seagar AL, Holden MTG, Laurenson IF. Investigation of two cases of *Mycobacterium chelonae* infection in haemato-oncology patients using whole-genome sequencing and a potential link to the hospital water supply. J Hosp Infect 2021;114:111–116. 33945838 10.1016/j.jhin.2021.04.028

[8] Gross JE, Caceres S, Poch K, et al. Prospective healthcare-associated links in transmission of nontuberculous mycobacteria among people with cystic fibrosis (pHALT NTM) study: Rationale and study design. PLoS One 2023;18:e0291910. 38117792 10.1371/journal.pone.0291910 PMC10732400

[9] Gross JE, Caceres S, Poch K, et al. Healthcare-associated links in transmission of nontuberculous mycobacteria among people with cystic fibrosis (HALT NTM) study: Rationale and study design. PLoS One 2021;16:e0261628. 34929010 10.1371/journal.pone.0261628 PMC8687591

[10] U.S. Green Building Council. LEED rating system. 2023. https://www.usgbc.org/leed Accessed March 20, 2025.

[11] Epperson LE, Strong M. A scalable, efficient, and safe method to prepare high quality DNA from mycobacteria and other challenging cells. J Clin Tuberc Other Mycobact Dis 2020;19:100150. 32154387 10.1016/j.jctube.2020.100150 PMC7052505

[12] Gross JE, Caceres S, Poch K, et al. Investigating nontuberculous mycobacteria transmission at the Colorado adult cystic fibrosis program. Am J Respir Crit Care Med 2022;205:1064–1074. 35085056 10.1164/rccm.202108-1911OC PMC9851486

[13] Honda JR, Hasan NA, Davidson RM, et al. Environmental nontuberculous mycobacteria in the Hawaiian Islands. PLoS Negl Trop Dis 2016;10:e0005068. 27780201 10.1371/journal.pntd.0005068 PMC5079566

[14] Virdi R, Lowe ME, Norton GJ, et al. Lower recovery of nontuberculous mycobacteria from outdoor Hawai’i Environmental water biofilms compared to indoor samples. Microorganisms 2021;9:224. 33499212 10.3390/microorganisms9020224 PMC7910870

[15] Adekambi T, Drancourt M, Raoult D. The rpoB gene as a tool for clinical microbiologists. Trends Microbiol 2009;17:37–45. 19081723 10.1016/j.tim.2008.09.008

[16] Walters W, Hyde ER, Berg-Lyons D, et al. Improved bacterial 16S rRNA Gene (V4 and V4-5) and fungal internal transcribed spacer marker gene primers for microbial community surveys. mSystems 2016;1:10–1128. 10.1128/mSystems.00009-15 PMC5069754 27822518

[17] Davidson RM, Nick SE, Kammlade SM, et al. Genomic analysis of a hospital-associated outbreak of *Mycobacterium abscessus*: Implications on transmission. J Clin Microbiol 2022;60:e0154721. 34705540 10.1128/JCM.01547-21 PMC8769749

[18] Gross JE, Teneback CC, Sweet JG, et al. Molecular epidemiologic investigation of *Mycobacterium intracellulare* subspecies *chimaera* lung infections at an adult cystic fibrosis program. Ann Am Thorac Soc 2023;20:677–686. 36656594 10.1513/AnnalsATS.202209-779OC PMC10174128

[19] Tamura K, Stecher G, Kumar S. MEGA11: Molecular evolutionary genetics analysis version 11. Mol Biol Evol 2021;38:3022–3027. 33892491 10.1093/molbev/msab120 PMC8233496

[20] Xu S, Li L, Luo X, Chen M, Tang W, Zhan L, Dai Z, Lam TT, Guan Y, Yu G. *Ggtree*: A serialized data object for visualization of a phylogenetic tree and annotation data. Imeta 2022;1:e56. 38867905 10.1002/imt2.56 PMC10989815

[21] Sehulster L, Chinn RYW, Arduino MJ, et al. Guidelines for environmental infection control in health-care facilities. Recommendations of CDC and the Healthcare Infection Control Practices Advisory Committee (HICPAC). MMWR 2003;52:1–48. 12836624

[22] American Society of Heating, Refrigerating and Air-Conditioning Engineers (ASHRAE). ANSI/ASHRAE Standard 514-2023: Risk Management for Building Water Systems: Physical, Chemical, and Microbial Hazards. Washington DC: American National Standards Institute; 2023.

[23] NSF International. NSF/ANSI/CAN 60: Drinking Water Treatment Chemicals- Health Effects. Ann Arbor, MI: NSF International; 2023.

[24] Prabaker K, Muthiah C, Hayden MK, et al. Pseudo-outbreak of *Mycobacterium gordonae* following the opening of a newly constructed hospital at a Chicago Medical Center. Infect Control Hosp Epidemiol 2015;36:198–203. 25633003 10.1017/ice.2014.28

[25] Kessler MA, Osman F, Marx J, Jr , Pop-Vicas A, Safdar N. Hospital-acquired Legionella pneumonia outbreak at an academic medical center: Lessons learned. Am J Infect Control 2021;49:1014–1020. 33631307 10.1016/j.ajic.2021.02.013

[26] World Health Organization. *WHO Guidance for Climate Resilient and Environmentally Sustainable Health Care Facilities*. 2020. https://www.who.int/publications/i/item/9789240012226 Accessed March 20, 2025.

[27] Dowdell KS, Potgieter SC, Olsen K, et al. Source-to-tap investigation of the occurrence of nontuberculous mycobacteria in a full-scale chloraminated drinking water system. Appl Environ Microbiol 2024;90:e0060924. 39109876 10.1128/aem.00609-24 PMC11409651

[28] LeChevallier MW, Prosser T, Stevens M. Opportunistic pathogens in drinking water distribution systems- a review. Microorganisms 2024;12:916 38792751 10.3390/microorganisms12050916 PMC11124194

[29] Rhoads WJ, Pruden A, Edwards MA. Survey of green building water systems reveals elevated water age and water quality concerns. Environ Sci Water Res Technol 2016;2:164–173.

[30] American Water Works Association. *Effects of Water Age on Distribution System Water Quality*. Prepared for the U.S. Environmental Protection Agency, Office of Ground Water and Drinking Water, Standards and Risk Management Division. August 15, 2002.

[31] D’Antonio S, Rogliani P, Paone G, et al. An unusual outbreak of nontuberculous mycobacteria in hospital respiratory wards: Association with nontuberculous mycobacterial colonization of hospital water supply network. Int J Mycobacteriol 2016;5:244–247. 27242241 10.1016/j.ijmyco.2016.04.001

[32] Donohue MJ, Vesper S, Mistry J, Donohue JM. Impact of chlorine and chloramine on the detection and quantification of *Legionella pneumophila* and *Mycobacterium species* . Appl Environ Microbiol 2019;85:e01942–19. 31604766 10.1128/AEM.01942-19 PMC6881805

[33] Dowdell K, Haig SJ, Caverly LJ, Shen Y, LiPuma JJ, Raskin L. Nontuberculous mycobacteria in drinking water systems – the challenges of characterization and risk mitigation. Curr Opin Biotechnol 2019;57:127–136. 31003169 10.1016/j.copbio.2019.03.010 PMC6924000

[34] Falkinham JO, Norton CD, LeChevallier MW. Factors influencing numbers of *Mycobacterium avium, Mycobacterium intracellulare*, and other *Mycobacteria* in drinking water distribution systems. Appl Environ Microbiol 2001;67:1225–1231. 11229914 10.1128/AEM.67.3.1225-1231.2001 PMC92717

[35] Guspiel A, Menk J, Streifel A, et al. Management of risks from water and ice from ice machines for the very immunocompromised host: A process improvement project prompted by an outbreak of rapidly growing mycobacteria on a pediatric hematopoietic stem cell transplant (HSCT) unit. Infect Control Hosp Epidemiol 2017;38:792–800. 28532525 10.1017/ice.2017.73

[36] Iroh Tam PY, Kline S, Wagner JE, et al. Rapidly growing mycobacteria among pediatric hematopoietic cell transplant patients traced to the hospital water supply. Pediatr Infect Dis J 2014;33:1043–1046. 24781138 10.1097/INF.0000000000000391

[37] Baker AW, Stout JE, Anderson DJ, et al. Tap water avoidance decreases rates of hospital-onset pulmonary nontuberculous mycobacteria. Clin Infect Dis 2021;73:524–527. 32829397 10.1093/cid/ciaa1237 PMC8326555

[38] Engers DW, Swarup R, Morrin C, et al. A bronchoscopy-associated pseudo-outbreak of *Mycobacterium chelonae* and *Mycobacterium mucogenicum* associated with contaminated ice machine water and ice. Infect Control Hosp Epidemiol 2023;44:2056–2058. 37272469 10.1017/ice.2023.101

[39] Cazals M, Bedard E, Soucy C, Savard P, Prevost M. How clean is your ice machine? Revealing microbial amplification and presence of opportunistic pathogens in hospital ice-water machines. J Hosp Infect 2023;141:9–16. 37604277 10.1016/j.jhin.2023.08.007

[40] Millar BC, Moore JE. Hospital ice, ice machines, and water as sources of nontuberculous mycobacteria: Description of qualitative risk assessment models to determine host-Nontuberculous mycobacteria interplay. Int J Mycobacteriol 2020;9:347–362. 33323649 10.4103/ijmy.ijmy_179_20

[41] Von Reyn CF, Maslow JN, Barber TW, Falkinham JO, 3^rd^, Arbeit RD. Persistant colonisation of potable water as a source of *Mycobacterium avium* infection in AIDS. Lancet 1994;343:1137–1141. 7910236 10.1016/s0140-6736(94)90239-9

