# Supplementary figures and images for: Mycobacterium chelonae outbreak investigation at a quaternary pediatric hospital following the opening of a LEED-certified critical care tower: where does water sustainability intersect with infection control?
Source: Infect Control Hosp Epidemiol. 2025 Nov 24;47(2):161–9. doi: 10.1017/ice.2025.10344 (PMC12926336; doi:10.1017/ice.2025.10344)

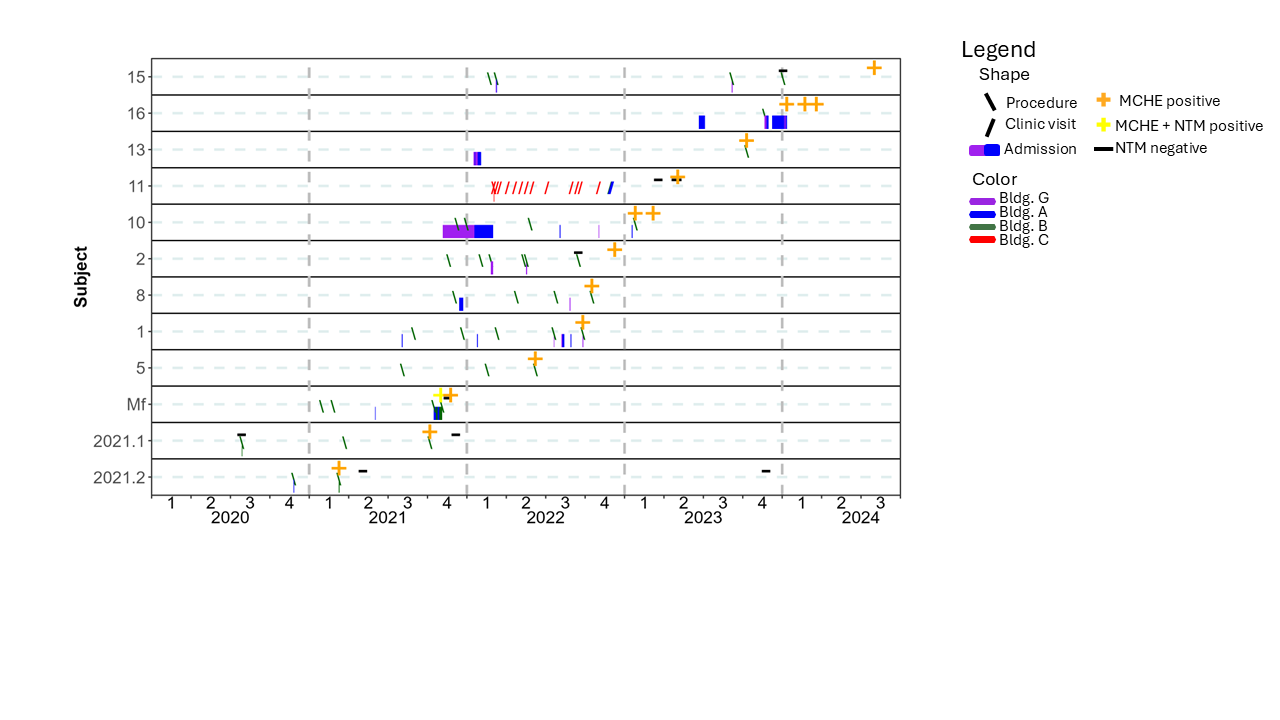

Supplement: Ankrum et al. supplementary material [file S0899823X25103449sup001.tiff]
